# Clinical validation for automated geographic atrophy monitoring on OCT under complement inhibitory treatment

**DOI:** 10.1038/s41598-023-34139-2

**Published:** 2023-04-29

**Authors:** Julia Mai, Dmitrii Lachinov, Sophie Riedl, Gregor S. Reiter, Wolf-Dieter Vogl, Hrvoje Bogunovic, Ursula Schmidt-Erfurth

**Affiliations:** 1 Laboratory for Ophthalmic Image Analysis (OPTIMA), Department of Ophthalmology and Optometry, Medical University of Vienna, Währinger Gürtel 18-20, 1090 Vienna, Austria; 2 Christian Doppler Laboratory for Artificial Intelligence in Retina, Department of Ophthalmology and Optometry, Medical University of Vienna, Vienna, Austria

**Keywords:** Medical research, Medical imaging

## Abstract

Geographic atrophy (GA) represents a late stage of age-related macular degeneration, which leads to irreversible vision loss. With the first successful therapeutic approach, namely complement inhibition, huge numbers of patients will have to be monitored regularly. Given these perspectives, a strong need for automated GA segmentation has evolved. The main purpose of this study was the clinical validation of an artificial intelligence (AI)-based algorithm to segment a topographic 2D GA area on a 3D optical coherence tomography (OCT) volume, and to evaluate its potential for AI-based monitoring of GA progression under complement-targeted treatment. 100 GA patients from routine clinical care at the Medical University of Vienna for internal validation and 113 patients from the FILLY phase 2 clinical trial for external validation were included. Mean Dice Similarity Coefficient (DSC) was 0.86 ± 0.12 and 0.91 ± 0.05 for total GA area on the internal and external validation, respectively. Mean DSC for the GA growth area at month 12 on the external test set was 0.46 ± 0.16. Importantly, the automated segmentation by the algorithm corresponded to the outcome of the original FILLY trial measured manually on fundus autofluorescence. The proposed AI approach can reliably segment GA area on OCT with high accuracy. The availability of such tools represents an important step towards AI-based monitoring of GA progression under treatment on OCT for clinical management as well as regulatory trials.

## Introduction

Due to a progressively ageing population, the number of patients diagnosed with geographic atrophy (GA) secondary to age-related macular degeneration (AMD) is expected to grow steadily^1^. Given the increasing burden of this disease, treatment for GA secondary to AMD is highly sought after, which is reflected by the high number of interventional clinical trials in the field. Several phase 2 up to phase 3 clinical trials are ongoing or have been completed to investigate potential new treatments^2,3^. The phase 2 FILLY clinical trial (NCT02503332), which studied the therapeutic efficacy of pegcetacoplan, a complement C3-inhibitor, revealed promising results by showing a significantly decreased GA growth rate in treated patients compared to sham patients after 1 year^4^. This led to two phase 3 studies DERBY (NCT03525600) and OAKS (NCT03525613) and pegcetacoplan has now been approved as the first therapy for GA secondary to AMD.

In clinical trials anatomic endpoints like GA growth rate are used to evaluate the efficacy of new treatments^5,6^, also done so in the FILLY trial. The current standard imaging modality in clinical trials evaluating therapeutic efficacy on GA progression is fundus autofluorescence (FAF)^6^. In clinical practice, however, optical coherence tomography (OCT) is more widely available and is established as standard of care for the monitoring of AMD, especially exudative AMD^7^. As spectral-domain OCT (SD-OCT) generates 3D volumes composed of a set of 2D cross-sectional B-scans, it offers more detailed information about the morphology of the affected retinal layers. This provides a detailed understanding of pathomorphologic changes in GA on the level of the neurosensory layers^8^. In particular, early morphological changes such as photoreceptor degeneration can only be seen reliably on OCT^9,10^. Photoreceptor degeneration at the margins of the GA lesion, the junctional zone, have been shown to be early indicators of disease progression^11–13^. Visual acuity can be preserved until an advanced stage of the disease, until the atrophy progresses to the foveal tissue^14^. The assessment and quantification of foveal involvement is superior on OCT compared to blue-light FAF^15^, leading to a superior estimate of the visual acuity preservation. As soon as the fovea is involved, visual acuity is strongly and irreversibly reduced. Therefore, foveal involvement represents a critical stage during the course of the disease, and the prevention of foveal involvement is a promising target for new treatments. Now that a treatment for GA has become available, huge numbers of patients will have to be monitored with respect to the assessment of treatment indication and evaluation of a potential therapeutic benefit in slowing down the disease progression. Given these perspectives, a strong need for automated GA segmentation has evolved. Image analysis methods using artificial intelligence (AI) are suitable to be used to process large amounts of data in a fast, accurate and reproducible way, thus saving labor and cost-intensive human resources^16^.

In this study, we developed and evaluated a novel fully-automated deep learning-based algorithm for GA segmentation and area quantification on OCT using real-world data from routine clinical care. We further validated the algorithm on an independent external validation set from a clinical trial and the performance of the algorithm was compared to the inter-grader variability of two experienced graders. Additionally, we evaluated the potential of the algorithm for monitoring GA progression under complement inhibitory therapy with OCT.

## Methods

### Data sets and study population

The study population consisted of two independent cohorts of SD-OCT scans and FAF images (both Spectralis, Heidelberg Engineering, Heidelberg, Germany) of GA patients, one originating from the Medical University of Vienna (MUV) and the other from the randomized sham-controlled FILLY phase 2 trial. Only Spectralis scans were included for this analysis due to the higher image quality (signal to noise ratio) and because follow-up scan acquisition was anatomically aligned, facilitating the topographic growth evaluation.

The real-world GA cohort from the MUV consisted of 184 eyes of 100 consecutive patients from routine clinical practice, who were 50 years of age or older and had a diagnosis of GA secondary to non-neovascular AMD on FAF images, assessed by two experienced graders. A detailed description has been published previously^17^. In short, patients were excluded if there was a history of other ocular diseases that would confound retinal assessment. Patients were followed-up every 3 months for at least 12 months, and FAF and SD-OCT scans (20° × 20°, resolution of 1024 × 49 A-scans × B-scans, ART 9) were performed at every visit. FAF images were excluded in case of insufficient image quality, preventing accurate measurement of atrophy size.

The FILLY trial was a randomized sham-controlled phase 2 clinical study of intravitreal pegcetacoplan, an investigational therapy targeting complement C3, for GA secondary to AMD (NCT02503332). The detailed inclusion and exclusion criteria have been published previously^4^. In brief, 246 patients were recruited in the trial, which were randomized in a 2:2:1:1 manner to receive either 15 mg intravitreal pegcetacoplan monthly (AM), every other month (AEOM) or a sham injection monthly or every other month (SM). The imaging data consisted of OCT scans, taken in a raster of 512 × 49 × 496 voxels, Infrared Reflectance (IR), and FAF images. The primary outcome measure was the change in square root GA lesion size from baseline to month 12 assessed by a centralized reading center on FAF images, using a semi-automatic region finder software tool^18^. Patients were excluded if there was presence of GA secondary to causes other than AMD, history or current evidence of exudative AMD at screening, and retinal disease other than AMD.

The presented study was approved by the Ethics Committee of the MUV (EK Nr: 1246/2016). The research was performed in compliance with the tenets of the Declaration of Helsinki and Good Clinical Practice. Written informed consent was given by all patients before any study-specific procedure.

### Image annotation

#### OCT annotation of the real-world cohort

The FAF images from the MUV cohort were annotated manually by delineating the GA area, defined as well-demarcated areas with a significantly decreased or extinguished degree of autofluorescence. The grading was done by two trained graders, using a validated image analysis software (OCTAVO, Vienna Reading Center, Vienna, Austria). To obtain matched OCT gradings, the annotated FAF images were automatically anatomically registered to the corresponding near infrared reflectance (NIR) image, which were aligned with the acquired OCT scans by the imaging device, resulting in 2D en-face OCT annotations. A deep learning-based spatial registration method^19^ was employed, and the registration process corrected the difference in magnification between the FAF image and the OCT scan.

#### OCT annotation of the study cohort

The OCT scans from study eyes of the FILLY trial were assessed by one trained image annotator from a group of four at the Ophthalmic Image Analysis (OPTIMA) research group in Vienna and supervised by an experienced clinician. Complete retinal pigment epithelium (RPE) loss was manually annotated on whole OCT volumes obtained at baseline and month 12. RPE loss was defined as the complete absence of RPE in combination with a hypertransmission in the underlying choriocapillaris without minimum size requirements. Annotations were performed on an A-scan level using an in-house software tool, delineating the areas of the RPE loss on every OCT B-scan. An example of an OCT B-scan with manual annotation is provided in Supplementary Fig. 1. To investigate inter-grader variability, the baseline OCT volumes were split into quartiles by respective GA size. From each quartile, 3 volumes were randomly selected, and the resulting 12 OCT volumes were annotated and supervised by another experienced clinician. OCT volumes with insufficient image quality to allow manual annotation as well as patients that did not complete follow-up at month 12 were excluded from this analysis.

### Development of the deep-learning model

A deep neural network architecture for 3D-to-2D image segmentation, first introduced by Lachinov et al.^20^, was employed to train an automated image segmentation model to delineate 2D en-face GA areas from a set of annotated 3D OCT volumes. The method takes entire OCT volumes as an input, benefiting from spatial 3D context, and provides the likelihood of atrophic changes to be present in each A-scan in the form of a 2D en-face map. The model was trained on 3D patches and corresponding en-face reference masks randomly sampled from OCT volumes. To segment a single A-scan, the method evaluates the spatial context of 1.09 × 1.03 mm^2^. Fully convolutional nature of the model enables parallel processing of the A-scans, taking a few seconds to process a single OCT volume. Details on the development of the model are provided in the Supplementary Material.

### Training, validation and external testing

For training and internal validation, we employed a fivefold cross-validation setup. The MUV dataset was split into five groups at the patient level with stratification by the baseline lesion size. Five instances of our segmentation model were trained. For each instance, one group was selected as validation set and the remaining four groups as training set. After the training, each model processed the corresponding validation set. The cross-validation scheme allows to anticipate the performance of the model on the unseen data. Having multiple slightly different models in addition to providing more robust solutions also allows estimating the segmentation uncertainty as the standard deviation across the models. The results from the five validation sets were pooled together and the segmentation performance metrics described below were computed.

For external testing, the previously unseen FILLY dataset was employed to get an unbiased estimate of what the performance would be on an independent dataset^21^. Each OCT volume was processed by five trained models and their output segmentation masks were averaged. The averaging was performed by calculating the mean across predictions of the models. The segmentation performance metrics were then computed on the final averaged segmentation masks. Details on the training of the model are provided in the Supplementary Material.

The following evaluations of the model performance were conducted. (i) The automated segmentation of GA was evaluated on the baseline and month 12 OCT scans of the internal validation set and of the external test set by comparing them to the respective manual annotations. In addition, in a subset of external test scans, the automated measurements were compared to the inter-grader variability. (ii) The automated segmentation of the GA growth area at month 12 was compared to the manually annotated area on the external test set. (iii) The growth rates obtained from automated processing of OCT scans of the FILLY trial were compared to the growth rates assessed manually on OCT, and the difference in OCT-based growth rates between the treatment arms of the FILLY trial were statistically analysed.

### Statistical analysis

The performance of the algorithm was evaluated by calculating the mean ± standard deviation (SD) and median with interquartile range (IQR) of the Dice Similarity Coefficient (DSC), representing the overlap between the segmented GA topographic area and the manual expert annotation. As the DSC has its limitation in the evaluation of small lesion areas, we computed the Hausdorff distance (HD) as additional evaluation metric to detect the presence of local outliers in the segmented lesion contour. For instance in regions that were wrongly segmented as GA, the DSC could result in a high score if the overall overlap is big but won’t spot the outlier. This metric indicates the quality of the lesion localization to provide an overlap-based metric (DSC) and distance-based metric (HD) to have a comprehensive evaluation^22^. Since the Hausdorff distance is especially sensitive to outliers, instead of taking the maximum distance between segmented and ground-truth surfaces, 95 percentile was calculated. The inter-grader variability was evaluated by calculating the DSC of the two manually annotated areas and the intraclass correlation coefficient (ICC) using a two-way random effects model. Corresponding 95% confidence intervals (CI) were calculated following Shrout et al.^23^.

For the 12-month growth area, mean ± SD and median [IQR] DSC and HD were computed. The GA growth rate was defined as the difference between the GA area at baseline and month 12. To adjust for baseline lesion size, the GA growth rate was calculated using the previously established square root transformation by calculating the difference between the square root transformed GA growth areas of month 12 and baseline, respectively^24^. The correlation between the growth rates of manual annotation and automated segmentation for the different treatment groups was reported using Pearson’s correlation coefficient r and the coefficient of determination R^2^. All statistical analyses were done using Python and its packages scipy, medpy and pingouin.

### Conference presentation

This work was in parts presented at the Annual Meeting of the Association of Research in Vision and Ophthalmology 2021.

## Results

### Data set and baseline characteristics

For training and internal validation, 967 OCT volumes (at all time points) from 184 study eyes of 100 patients were used from the MUV GA cohort. For the external test set, 226 OCT volumes (baseline + month 12) were used from 113 study eyes of 113 patients from the FILLY trial. Detailed information on patient numbers and excluded data is shown as flowchart in Supplementary Fig. 2. In total, 11,074 B-scans of the external test set were annotated manually. The baseline characteristics of the study population are summarized in Table 1. Both datasets showed comparable age and gender distributions.

**Table 1:** Baseline characteristics of the study population for internal (MUV) and external (FILLY) test set. Baseline GA area and GA growth rate are presented as square root transformed values. *Calculated as the mean of all treatment groups.

Data type	MUV	FILLY
Number of patients	100	113
Baseline age (years), mean (SD)	75.8 (7.4)	78.9 (7.2)
Female gender, no. (%)	64 (65.2)	74 (65.5)
Baseline GA area (mm), mean (95% CI)	2.44 (2.28–2.58)	2.61 (2.48–2.73)
GA growth rate (mm/y), mean (95% CI)	0.32 (0.28–0.36)	0.23 (0.20–0.27)*

### AI performance evaluation

The performance of the automated GA segmentation from the internal and external test sets at baseline and month 12 was evaluated using the DSC. Results are presented as mean ± SD. In the internal test set, mean DSC was 0.82 ± 0.15 for baseline and 0.87 ± 0.11 for month 12 GA area. Mean overall DSC for all scans was 0.86 ± 0.12 in the internal test set. In the external test set, the performance of the automated segmentation was numerically higher than in the internal test set with a mean DSC of 0.91 ± 0.05 for baseline and 0.92 ± 0.05 for month 12 GA area. Mean overall DSC for all scans was 0.91 ± 0.05 in the external test set. Detailed evaluation metrics for internal and external validation are presented in Table 2 and Supplementary Table 1. The distribution of the DSC and HD95 for baseline and month 12 GA area as well as the distribution of DSC across different GA lesion sizes at baseline is presented in the Supplementary Figs. 3, 4. The algorithm performed well for smaller as well as for larger GA lesion sizes.

**Table 2:** Evaluation metrics of the algorithm for internal and external validation and inter-grader variability. *DSC* Dice Similarity Coefficient, *SD* standard deviation, *IQR* interquartile range, *HD* Hausdorff distance (mm), *ICC* intraclass correlation coefficient, *G1* Grader 1, *G2* Grader 2, *P* prediction.

	N	DSC mean ± SD	DSC median [IQR]	HD95 mean ± SD	HD95 median [IQR]
Internal validation (MUV)					
Baseline	121	0.82 ± 0.15	0.85 [0.80; 0.92]	0.51 ± 0.42	0.38 [0.24; 0.65]
Month 12	121	0.87 ± 0.11	0.90 [0.85; 0.93]	0.48 ± 0.40	0.37 [0.21; 0.61]
All	967	0.86 ± 0.12	0.90 [0.84; 0.93]	0.54 ± 0.45	0.40 [0.24; 0.71]
External validation (FILLY)					
Baseline	113	0.91 ± 0.05	0.92 [0.89; 0.95]	0.40 ± 0.45	0.24 [0.13; 0.48]
Month 12	113	0.92 ± 0.05	0.93 [0.89; 0.95]	0.39 ± 0.36	0.26 [0.14; 0.46]
All	226	0.91 ± 0.05	0.93 [0.89; 0.95]	0.38 ± 0.40	0.24 [0.13; 0.44]

	N	DSC mean ± SD	ICC (95% CI)		
Inter-grader variability					
G1 vs. G2	12	0.92 ± 0.08	0.98 (0.93–0.99)		
P vs. G1	12	0.87 ± 0.10	0.98 (0.94–1.0)		
P vs. G2	12	0.90 ± 0.07	0.99 (0.96–1.0)		

**Figure 1:**
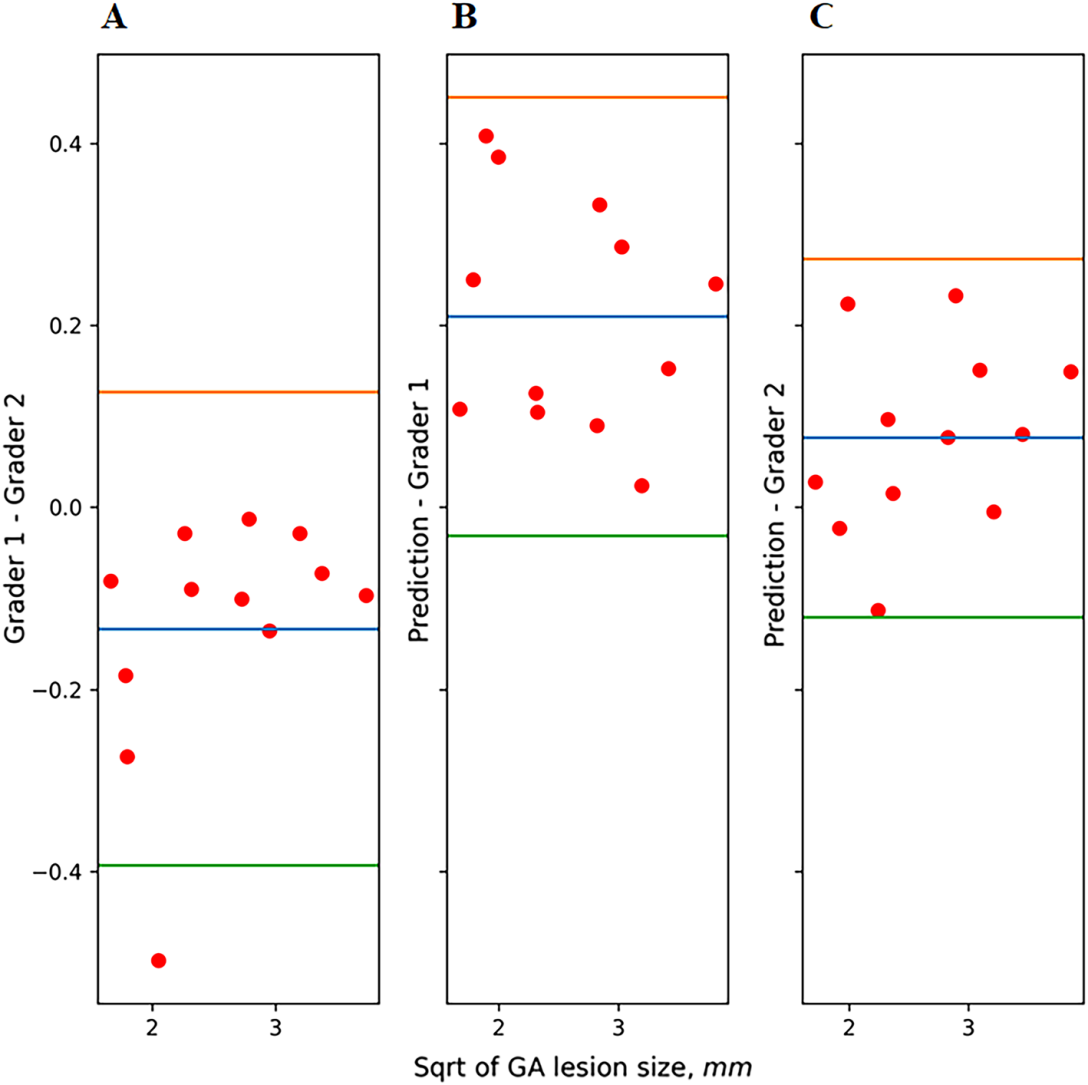
Limits of agreement for inter-grader agreement (**A**) and model-grader (**B**,**C**) agreements of the total geographic atrophy lesion size. The mean difference is plotted in blue and the limits of agreement are plotted in orange (mean difference + 1.96 standard deviation of the difference) and green (mean difference − 1.96 standard deviation of the difference). *Sqrt* square root transformation. Grader 1 slightly underestimated GA lesion size compared to grader 2. The model performance was closer to grader 2 than to grader 1. The limits of agreement were wider for the inter-grader agreement than for the model-grader agreements, showing that the AI model operated within inter-grader variability.

The results of the inter-grader variability on a subset of 12 OCT volumes are also shown in Table 2. Mean DSC and ICC were high between expert gradings as well as for model-grader agreements. Grader 1 slightly underestimated GA lesion size compared to grader 2. The limits of agreement were wider for the inter-grader agreement than for the model-grader agreements (Fig. 1), showing that the model operated within inter-grader variability.

**Figure 2:**
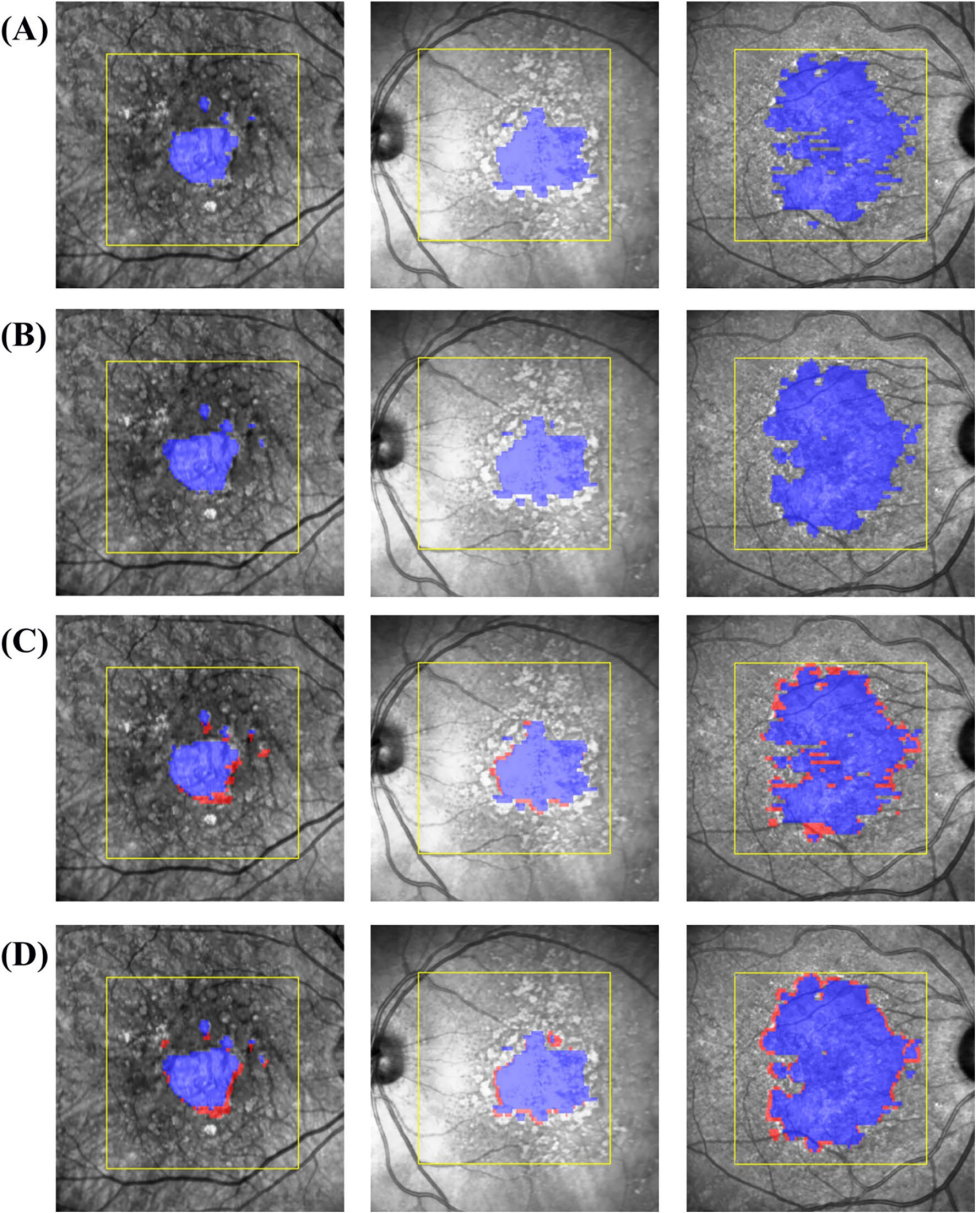
Examples of en-face segmentation of geographic atrophy on OCT of a small (first column), medium (second column) and large (third column) baseline lesion size, marked in blue. (**A**) represents the manually annotated baseline area, (**B**) represents the automatically segmented baseline area, (**C**) represents the manually segmented growth area at month 12, marked in red and (**D**) represents the automatically segmented growth area at month 12, marked in red.

To evaluate the performance of the algorithm for the segmentation of the GA growth area, the automated segmentation of the 12-month GA growth area was compared to the manual annotation on the external test set for all treatment groups pooled. Examples of the automatically segmented baseline GA area and 12-month growth area by the model are shown in Fig. 2. Mean DSC for segmenting the 12-month GA growth area on the external test set was 0.46 ± 0.16 and mean HD95 was 0.40 ± 0.36. The distribution of the DSC and HD95 for the GA growth area as well as the distribution of the DSC across GA lesion sizes at baseline is presented in the Supplementary Material (Supplementary Figs. 5, 6).

### AI-based monitoring of GA progression under therapy

**Figure 3:**
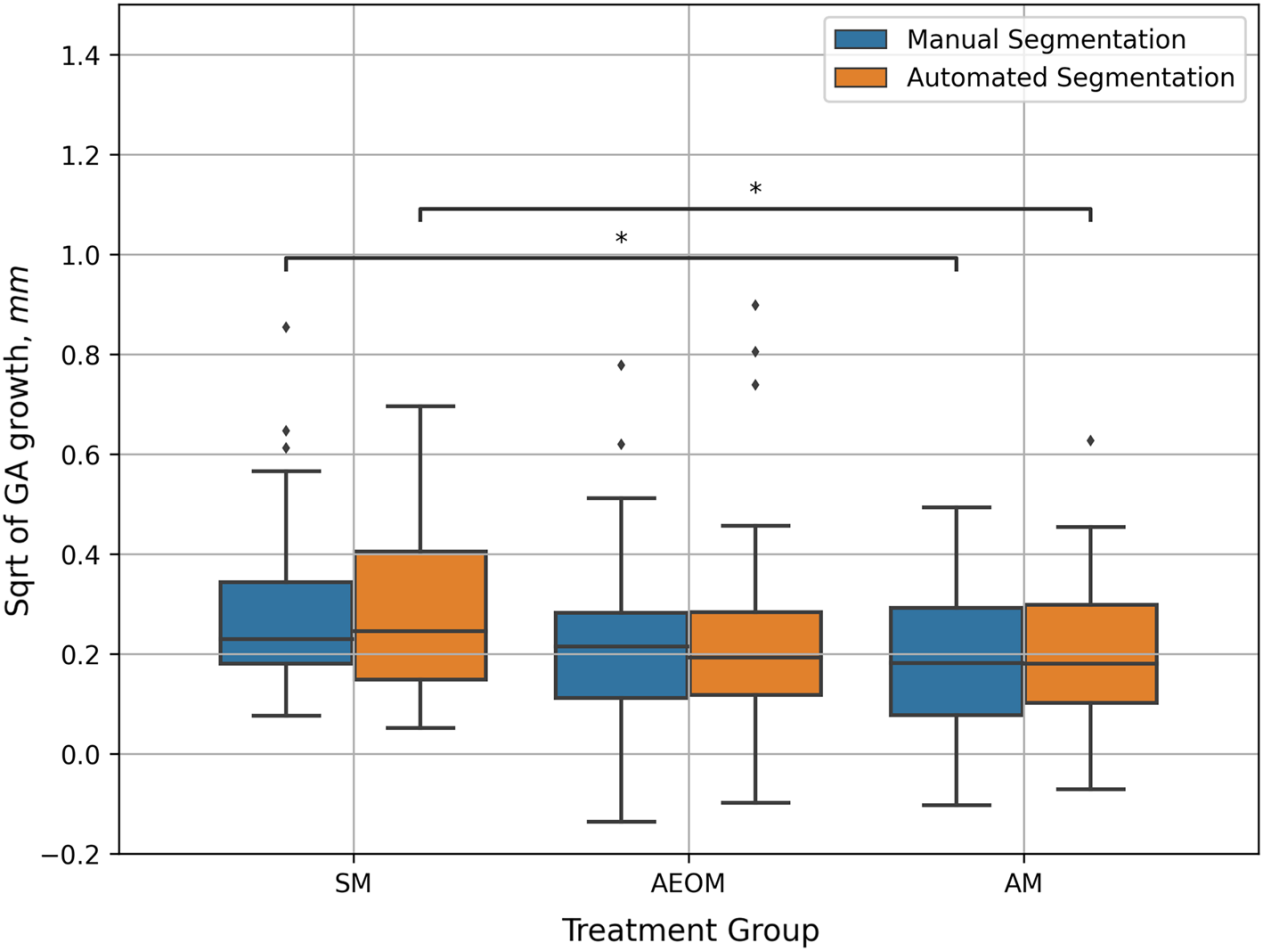
Boxplots for manual vs. automated segmentation of geographic atrophy growth on OCT at month 12 for the different treatment groups. Asterisks denote statistically significant difference in growth rates between SM and AM treatment group by automated segmentation (p = 0.030) and manual segmentation (p = 0.028). Note that there was no statistically significant difference between manual and automated segmented growth rates for all treatment groups. *SM* sham, *AEOM* every other month, *AM* monthly treated group, *Sqrt* square root transformation, *GA* geographic atrophy.

**Figure 4:**
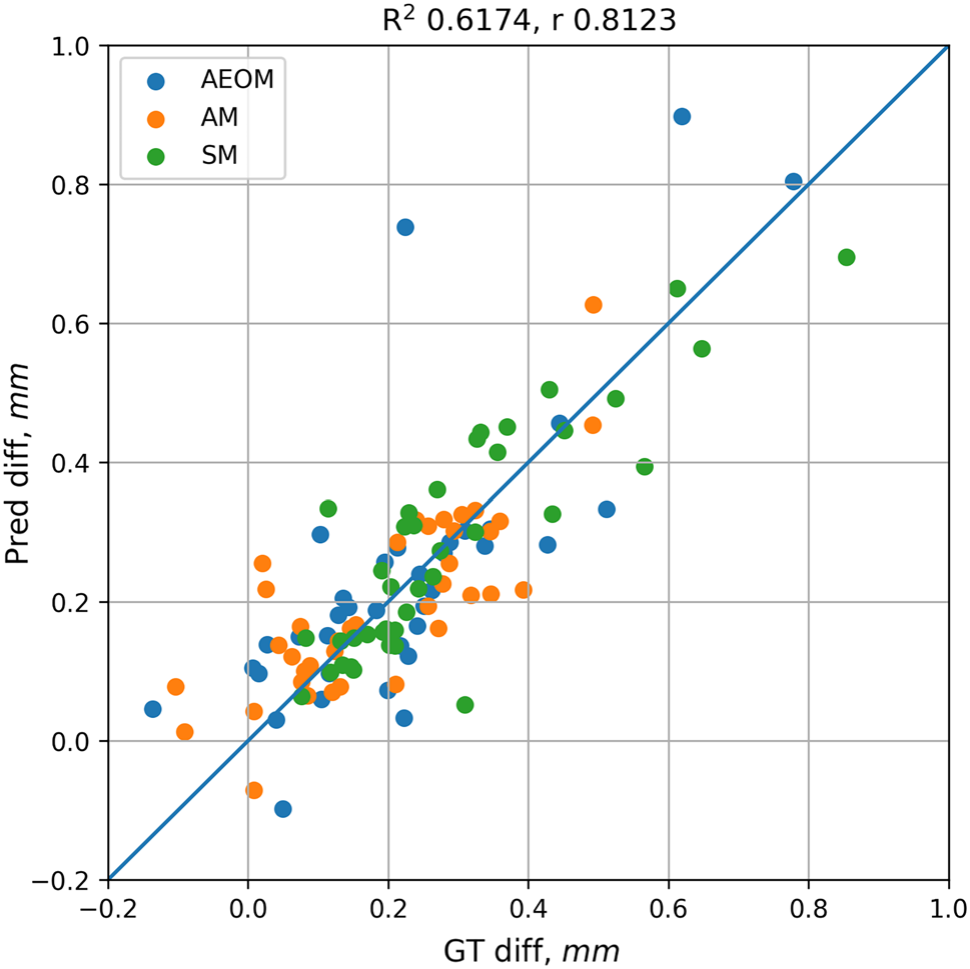
Correlation scatterplot for manual and predicted GA growth rates at month 12 for the different treatment groups. The correlation coefficient across treatment groups was r = 0.81 with an R^2^ = 0.62. *AEOM* every other month, *AM* monthly treated group, *SM* sham group, *GA* geographic atrophy, *GT* ground truth.

To evaluate the ability of the algorithm for the segmentation of the GA progression under complement inhibitory treatment, the correlation between the growth rates of manual annotation and automated segmentation for the different treatment groups was analysed. Automated vs. manually segmented GA growth rates by month 12 for the different treatment groups are shown in Fig. 3. There were consistent results in measuring the mean GA growth rate under therapy between automated vs. manual segmentation with a mean square root transformed growth rate at month 12 of 0.28 mm vs. 0.29 mm in the SM group, 0.24 mm vs. 0.22 mm in the AEOM group and 0.20 mm vs. 0.19 mm in the AM group, respectively. There was no statistically significant difference observed between the automated and the manually segmented growth rates in all treatment groups (SM: p = 0.920, AEOM: p = 0.942, AM: p = 0.807). The Pearson’s correlation coefficient between manual and automated GA growth rates across the treatment groups was 0.81 with an R^2^ of 0.62 (Fig. 4).

The fully-automated measured GA growth rate on OCT by month 12 was slower in the AM compared to the SM group (AM: 0.20 ± 0,13 mm vs. SM: 0.28 ± 0.17 mm, p = 0.030). This was in line with the primary results from the FILLY trial, where a significant slower GA growth was shown in treated patients compared to sham, measured manually on FAF. The difference in our automated measured growth rates between the treatment groups AEOM vs. SM showed a trend towards slower growth in the AEOM group (AEOM: 0.24 ± 0.21 mm vs. SM: 0.28 ± 0.17 mm, p = 0.106).

## Discussion

With this work, we present a novel deep learning-based algorithm for GA segmentation and quantification on OCT with high and consistent performance. The algorithm was able to accurately segment GA areas on OCT with a mean DSC of 0.86 in the internal test set and a mean DSC of 0.91 in the external test set. Notably, our algorithm reached the same level in DSC as the inter-grader variability of manual segmentation by human expert graders obtained with extensive manpower efforts.

The DSC of the GA growth area between baseline and month 12 was close to 0.5 and therefore lower than that of the segmentation of the total GA area. As the GA growth area represents a small region in most cases, especially compared to the total GA areas, every wrongly segmented pixel—manually or automatically—has a large impact on the average DSC, generally leading to a lower DSC result. As complimentary performance metric we calculated the HD95, which corresponds to the maximum distance between 2D en-face contours of the reference and the segmented region. The mean HD95 for the GA growth area was 0.40 mm, meaning that 95% of the values had an error below 0.40 mm. It was within the range of the mean HD95 of the external validation set for the total GA area (0.38 mm), which indicates the quality of the predicted lesion localization^25^. Additionally, we evaluated the correlation between manually and automatically segmented growth rates between treatment groups. No statistically significant difference was detected between the automated and the manually segmented growth rates at month 12 with a correlation coefficient of 0.81, which is a clinically relevant finding with regard to providing reasonable automated GA monitoring under therapy.

Precise morphological monitoring of disease activity and therapeutic response is crucial in GA. Functional parameters like best corrected visual acuity (BCVA) that are used to evaluate disease progression and therapeutic response in other retinal diseases, do not reflect disease progression in GA and are thus unsuitable for disease monitoring. BCVA can be preserved until an advanced stage of the disease, when the foveal tissue gets affected by the atrophic process^14^. Currently, slowing the anatomical growth rate of GA is an accepted clinical trial endpoint^5^. This underlines the importance of precise and objective image analysis methods to measure morphologic parameters in GA patients, especially since a novel treatment has now been approved by regulatory authorities. AI-based image analysis tools are urgently needed to support physicians in clinical practice to handle the large amount of data and particularly the subclinical hallmarks at the population level generated by the huge number of patients affected by GA who may benefit from regular monitoring. In particular, the regulatory authorities such as the U.S. Food and Drug Administration (FDA) have documented their interest in considering anatomical endpoints when functional parameters are too variable^26^. On using OCT as a modality it was noted that a more automated measurement is more prone to provide better accuracy^26^. A first step towards this goal is the automated segmentation of the RPE loss area on OCT.

To this end, we developed an automated algorithm for RPE loss segmentation on OCT, whose technical framework has been introduced previously^20^. To the best of our knowledge, our study is the first which evaluated AI-based longitudinal assessment of GA growth under therapy. Our algorithm was evaluated for GA progression monitoring on OCT and, furthermore, compared to the results of a prospective phase 2 clinical trial of an investigational complement inhibition therapy for GA. There was a slight difference in performance between the internal and external test set (mean DSC 0.86 in the internal test set vs. mean DSC 0.91 in the external test set). Of note, the internal and external test set derive from two completely independent datasets. One explanation for the difference in performance between the two datasets could be the clinical study setting of the external test set (FILLY trial) with standardized good quality images versus the real-world cohort setting of the internal test set. Moreover, for the internal validation, OCT annotations derived from FAF images were used as training data vs. manual annotations on OCT for the external validation. There could be a slight misregistration of the OCT and FAF images, which could also be an explanation for the difference in performance. We could show that the difference in growth rates between the treatment groups from the trial, measured semi-automatically on FAF, reached the same results as our deep learning-based algorithm using OCT scans from a subset of patients. Thus, we can conclude that FAF and OCT as well as manual and automated segmentation on OCT resulted in the same clinical trial outcome. Notably, the algorithm performed within the intergrader variability between two experts. Therefore, we believe that the performance of the algorithm has a high validity for possible clinical use.

Different approaches for automated GA segmentation on various imaging modalities have been proposed previously^27^. Most studies predominantly focused on GA segmentation on 2D FAF with a DSC ranging from 0.83 to 0.89^28,29^. As SD-OCT has become the standard imaging method in AMD in the clinical setting^7^, more studies have now focused on GA segmentation on OCT^30–32^ with a DSC range of 0.81–0.87^27^.

In contrast to our algorithm, which was trained on the pathology itself, namely the annotated RPE loss, previous algorithms mainly focused on choroidal hypertransmission to detect GA on OCT, which is a secondary consequence of overlying tissue loss^33,34^. By processing an OCT projection image obtained from the region between RPE/Bruch membrane and the choroid, those methods achieved an average DSC of 0.87^33^ and 0.81^34^, respectively. However, choroidal hypertransmission alone is not sufficient to identify GA on OCT as it underlies great inter-individual variability and is dependent on image quality as well as on the overall signal level of the OCT volume^35^. The inhomogeneity of the hypertransmission signal is also referred to as bar code pattern making it difficult to consistently quantify small changes in cellular loss.

Other AI methods used single A-scans of the OCT as an input instead of a projection image, reaching a DSC of 0.87^30^ and 0.91^36^. By only using the OCT A-scans as input, the algorithm cannot learn from the full 3D contextual information. Our algorithm was specifically trained to delineate a topographic GA area on a 2D en-face map, using the whole 3D OCT volume as input and benefitting from a rich spatial 3D context. Moreover, the algorithm performs equally to human expert graders in an independent external test set and was evaluated using a clinically-relevant endpoint.

Recently, Zhang et al. published an algorithm for GA segmentation on OCT trained on the FILLY data^37^. They used the previously reported classification system proposed by the Classification of Atrophy Meeting (CAM) group for the description of earlier lesions in atrophic AMD on OCT, namely incomplete RPE and outer retinal atrophy (iRORA) and complete RPE and outer retinal atrophy (cRORA). There are three major criteria that have to be present to meet the definitions for these lesions: (1) region of hypertransmission, (2) RPE disruption or attenuation and (3) signs of photoreceptor degeneration, with (1) and (2) < 250 µm diameter representing iRORA and ≥ 250 µm diameter representing cRORA^38,39^. These definitions, however, still have to be validated and implemented in clinical practice as they have shown to underlie substantial inter-reader variability^40^. The CAM classification originates from pre-AI times when an accurate measurement of the extension of alteration in the different layers was not available and therefore represents rather a qualitative assessment. With high-precision measurement using AI tools a distinct grading of GA progression has become possible and enables monitoring of disease activity and therapeutic response in area change on a micron scale, replacing the gross distinction between i- and c-RORA. Particularly to detect morphologic changes preceding GA and investigate potential earlier targets for new treatments requires a resolution superior to 250 µm. During the advanced progression of GA disease, the photoreceptor status has been shown to exceed and precede RPE loss and could therefore identify patients at risk for faster progression^11,41^. The method proposed by Zhang et al. was in contrast to our work trained on the FILLY data and validated on clinical data and reached a mean DSC for RPE loss in the external test set of 0.87 ± 0.21^37^.

Another publication recently reported on a deep learning-based method using the RORA classification. The method reached a mean DSC of 0.88 ± 0.074 and 0.84 ± 0.076 compared to two separate graders in the external test set. However, the number of OCT scans in the external test set was very limited (18 OCT volumes)^35^.

The algorithm we proposed in this work goes beyond the previous methods by taking full 3D context into account as opposed to slice-by-slice segmentation. Moreover, it adds the clinically most relevant value of investigating AI-based monitoring of GA progression under complement inhibition therapy on OCT. Two other studies by our group used the proposed algorithm on RPE loss segmentation to investigate the therapeutic effect of pegcetacoplan on OCT but with distinct different purposes. The work of Riedl et al. focused on the inhibition of photoreceptor thickness and integrity loss^42^. The work of Vogl et al. investigated the local GA growth estimation^43^—both aspects were not evaluated in our study. Both mentioned publications did not include the extensive clinical validation as was done in this study. However, they used additional automated algorithms as we believe it is crucial to introduce automated OCT monitoring of GA to the community, specifically under therapeutic conditions. Our results suggest that the proposed algorithm can be used for objective, scalable and precise quantification of GA areas on OCT over time. To be clinically applicable, the robustness of the model across different imaging devices and different disease stages has to be investigated. Further prospective, randomized studies are needed to evaluate the ability of the algorithm to be implemented in clinical practice.

Now that a treatment for GA secondary to AMD has become available, we believe that automated and objective AI methods will be indispensable in the management of GA patients and OCT-based treatment guidance in routine clinical practice. Treatment effects in GA patients cannot be assessed by BCVA change as in exudative AMD, yet treatment will be invasive and long-term. Patients will have to be motivated to follow the continued regimen and payers may request proof of benefit. AI models can be used to predict disease progression and identify further biomarkers which are correlated with future GA growth, thus helping us to better understand the underlying pathomechanisms^44–46^. Furthermore, treatment requirements may be adapted on an individual patient level based on such predictions. In respect to the huge GA population to be treated, only automated fast and accurate AI-based evaluation, i.e. by mouse click will be efficient.

A limitation of this study is a possible selection bias due to the post-hoc analysis of a potential non-random subset from the FILLY trial. Furthermore, this also defines the minimal GA size defined by the inclusion criteria of the trial (lesions > 2.5 mm^2^). Although the MUV GA cohort consists of real-world patients from routine clinical care, patients were excluded if they had other retinal diseases to train the algorithm on GA patients secondary to non-neovascular AMD only, in concordance with inclusion criteria of currently ongoing treatment trials. Also, the model was trained and evaluated on Spectralis scans only. More studies are needed to investigate the performance of the algorithm on other OCT devices as well as mixed cases. Potential discrepancies with the topline results reported in FILLY could be due to FAF having a bigger field of view than OCT. Furthermore, the automated registration of FAF-based annotations to OCT might lead to some discrepancies. However, this is only the case for internal evaluation, while for external validation the high-level expert annotations on every B-scan of the whole OCT volume were taken as the reference, which is a great strength of this study. The performance of the algorithm was even slightly higher in the laboriously annotated external test set than in the internal evaluation, maybe due to the settings of a randomized clinical trial. While the overall correlation of GA growth rates was high between the manual and automated method for the different treatment groups, the prediction for one individual patient can still be challenging. More extensive phase 3 data is needed for further evaluation.

In conclusion, we propose a fully-automated segmentation method for reliable delineation and quantitative measurement of GA areas on SD-OCT, developed on a real-world cohort. The method was shown to be capable of monitoring GA progression under therapy with the first successful therapeutic approach, i.e. complement inhibition, a major unmet need for future personalized treatment of GA. The method represents an important step toward AI-based monitoring of GA patients in clinical practice on a large scale.

## Supplementary Information

Supplementary Information.

## Data Availability

Original data for this research were provided by Apellis Pharmaceuticals. Data that support the findings of this study are available upon reasonable request.
