# Supplementary material for: Clinical validation for automated geographic atrophy monitoring on OCT under complement inhibitory treatment
Source: Sci Rep. 2023 Apr 29;13:7028. doi: 10.1038/s41598-023-34139-2 (PMC10148818; doi:10.1038/s41598-023-34139-2)
Supplement: Supplementary file 1 — Supplementary Information. [file 41598_2023_34139_MOESM1_ESM.pdf]

Real-world development and external validation for automated geographic atrophy monitoring on OCT under complement inhibitory treatment

Julia Mai[1]; Dmitrii Lachinov[1,2]; Sophie Riedl[1]; Gregor S. Reiter[1]; Wolf-Dieter Vogl[1]; Hrvoje Bogunovic[1,2]; Ursula Schmidt-Erfurth[1]*

Affiliations:

[1] Laboratory for Ophthalmic Image Analysis (OPTIMA), Department of Ophthalmology and Optometry, Medical University of Vienna, Vienna, Austria

[2]Christian Doppler Laboratory for Artificial Intelligence in Retina, Department of Ophthalmology and Optometry, Medical University of Vienna, Vienna, Austria

Address for Correspondence:

Ursula Schmidt-Erfurth, MD

Professor and Chair, Department of Ophthalmology, Medical University of Vienna

Währinger Gürtel 18-20, 1090 Vienna, Austria

Tel.: +43 1 40400 79310; Fax: +43 1 40400 79320

Email: ursula.schmidt-erfurth@meduniwien.ac.at

Supplementary Methods

Development of the deep learning model

The model implementation was previously described in Lachinov et al. [1] The method is specifically designed to handle the problem of 3D → 2D segmentation. It follows a U-Net structure [2] and consists of encoder and decoder. The encoder network converts the input image into compressed latent representation. In contrast to U-Net, the decoder restores the compressed representation only in en-face dimensions, leaving A-scan dimension compressed. The encoder and decoder are linked together with a specially designed projective skip-connections that compresses the encoder feature maps in the A-scan dimension and concatenates them to the corresponding decoder feature map. The final linear layer classifies the A-scans into being atrophic or not.

The network is built using average pooling with kernel size 2x2x2 and stride 2x2x2, trilinear upsampling with the upsampling factor 2x1x2, residual blocks with full pre-activation [3] and Instance normalization. [4] As a preprocessing, training scans were unified and resampled to 121.1 x 5.68 μm^2 en-face pixel spacing. In addition, IOWA reference layer segmentation [5] of outer RPE was used for flattening and cropping a region of interest of size 128 pixels in the A-scan dimension.

Training

During model development, 5-Fold cross validation on the training set was used. The training dataset was split into 5 non-overlapping groups at patient level with stratification by the baseline lesion size. Five models were trained, using a single group as a test set and the rest as a training set for model's parameters optimization and hyperparameters tuning. Each training set was randomly split further into 90% training and 10% train-validation sets. The latter was used for the epoch selection with the highest train-validation DSC.

Data augmentation was employed to enrich training data. First, a randomly sampled image was normalized using z-score normalization per B-scan. Next, the training data was additionally augmented by using random region cropping of size 32 B-scans x 256 A-scans x 128 pixels. The image was mirrored along and across B-scans with probability 0.5. Intensity

based augmentations, like contrast augmentation randomly sampled from [0.4, 1.6] interval, and intensity shift randomly sampled from [-0.8;0.8] interval was also employed.

The model was implemented using PyTorch and trained on two Nvidia 3080 GPUs. For training, Adam optimizer was used for 30,000 iterations with batch size 8. The learning rate was set to 0.001 and decayed by a factor of ten at 20,000th iteration. These hyperparameters were selected based on model's performance on the train-validation splits of the MUV dataset. The trained network has 5.16 million parameters.

Segmentation Inference

First, the scan was resampled to abovementioned pixel spacing and then processed with z-score normalization per B-scan. The image was padded with minimum intensity value to the closest image size divisible by 8. Then the scan was processed with five deep learning model instances trained on different splits of the MUV GA dataset. If the inference scan was a part of the training split, the corresponding model was excluded from the ensemble. The inference was performed on CPU, without the need for expensive GPUs. For the scan of the typical size 49x1024x128 the inference takes 7 seconds and 7.46 GB of RAM. After the inference, we revert the padding and resample the image back to the original pixel spacing.

References

1. Lachinov D, Seeböck P, Mai J, Schmidt-Erfurth U, Bogunović H. *Projective Skip-Connections for Segmentation Along a Subset of Dimensions in Retinal OCT*. 2021.
2. Ronneberger O, Fischer P, Brox T. U-Net: Convolutional Networks for Biomedical Image Segmentation. 2015; Cham.
3. He K, Zhang X, Ren S, Sun J. Identity Mappings in Deep Residual Networks. 2016; Cham.
4. Ulyanov D, Vedaldi A, Lempitsky V. Instance Normalization: The Missing Ingredient for Fast Stylization. 2016:arXiv:1607.08022. https://ui.adsabs.harvard.edu/abs/2016arXiv160708022U. Accessed July 01, 2016.
5. Li K, Wu X, Chen DZ, Sonka M. Optimal surface segmentation in volumetric images--a graph-theoretic approach. *IEEE Trans Pattern Anal Mach Intell.* 2006;28(1):119-134.

Supplementary Figures

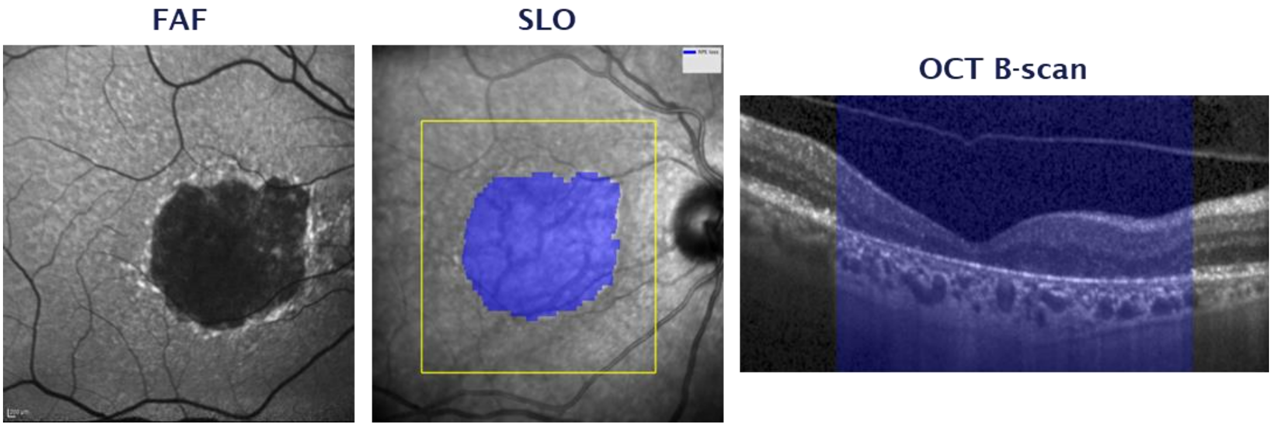

Supplementary Figure 1: Example of GA areas on FAF (left), SLO (middle) and manually annotated RPE loss (in blue) on central OCT B-scan (right). GA = geographic atrophy, FAF = fundus autofluorescence, SLO = scanning laser ophthalmoscopy, OCT = optical coherence tomography.

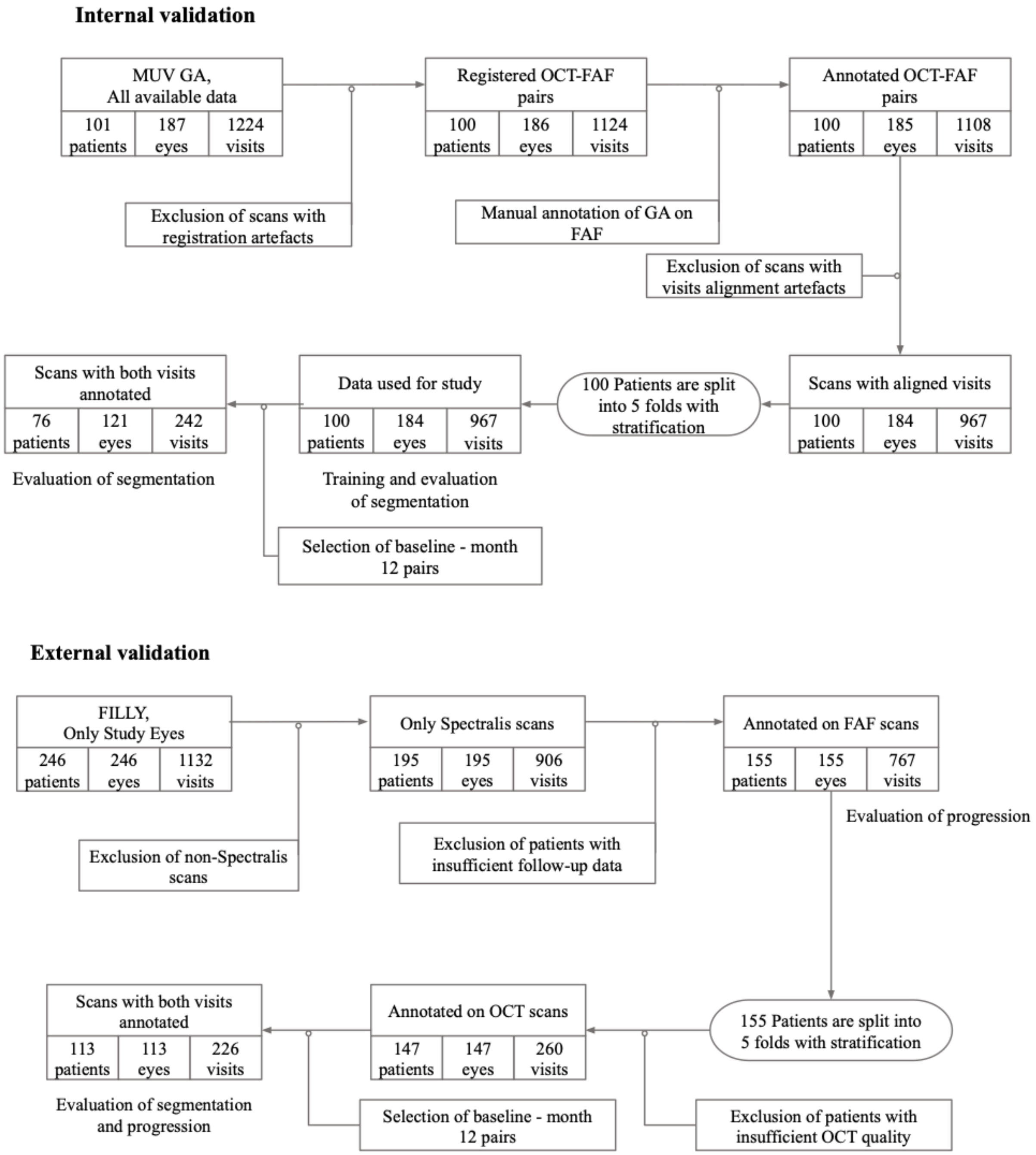

Supplementary Figure 2: Flowchart of study design.

The internal validation set consisted of OCT volumes from real-world patients with geographic atrophy from the Medical University of Vienna. The external validation set consisted of OCT volumes from patients with geographic atrophy from the FILLY trial with respective exclusion criteria shown in the figure.

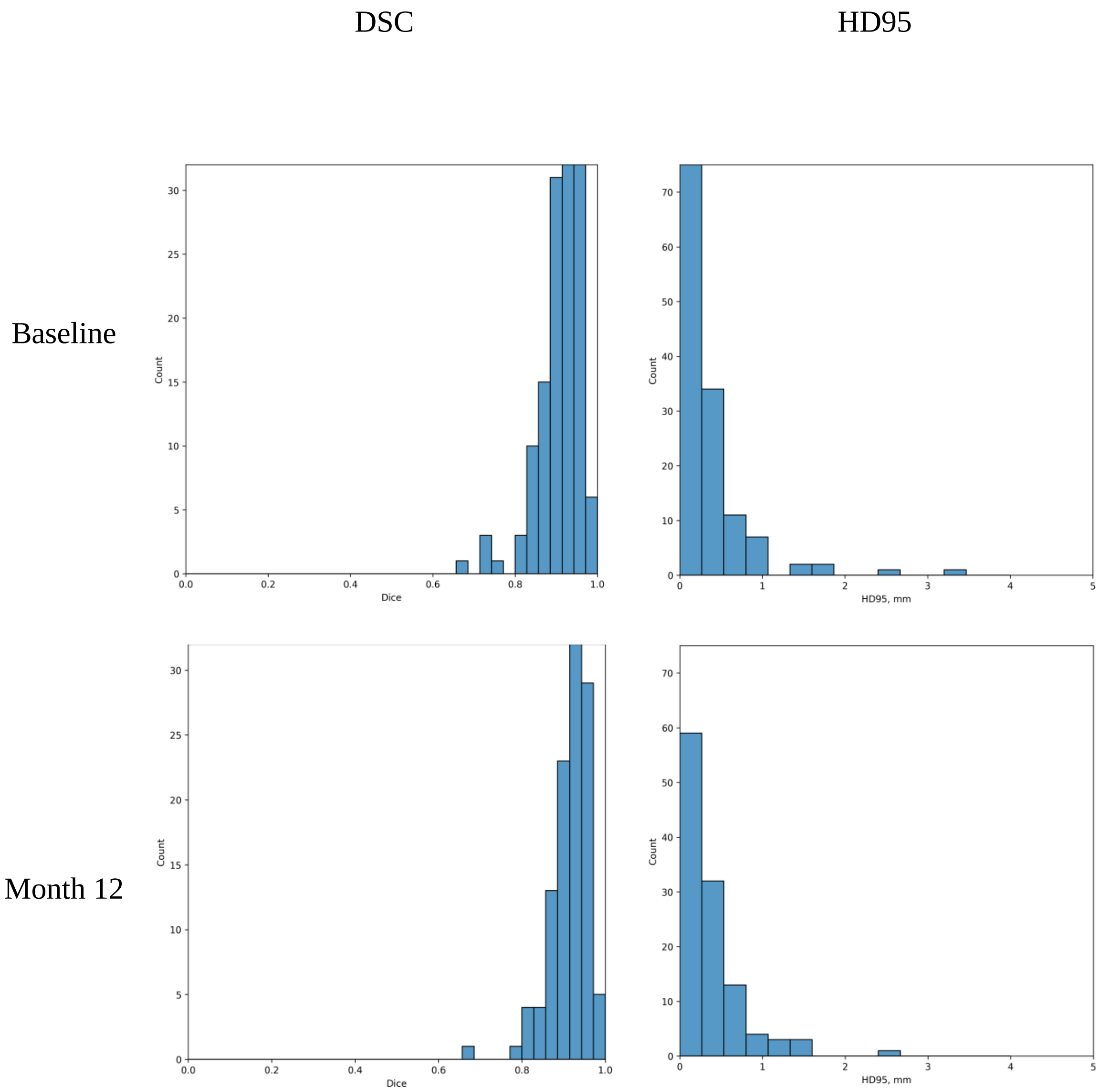

Supplementary Figure 3: Histogram of DSC and HD95 distribution of the deep learning model for RPE loss area at baseline (upper row) and month 12 (lower row) in the external test set. DSC = Dice Similarity Coefficient, HD = Hausdorff distance, GA = geographic atrophy.

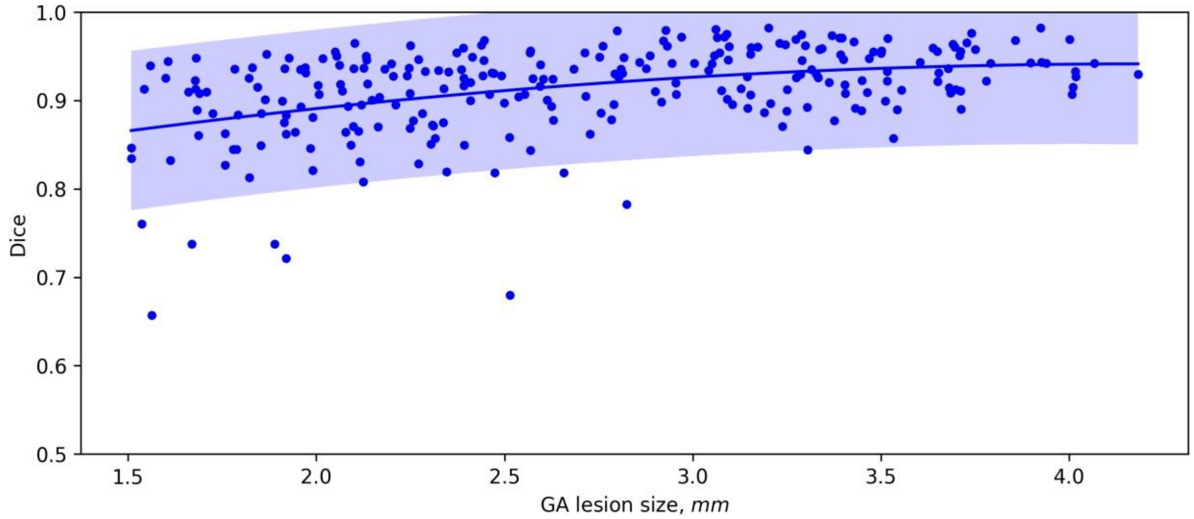

Supplementary Figure 4: Scatterplot for DSC distribution of the deep learning model for RPE loss across GA lesion sizes (square root transformed) at baseline. The blue stripe corresponds to the fitted gaussian process and indicates 95% limits of agreement. DSC = Dice Similarity Coefficient, GA = geographic atrophy.

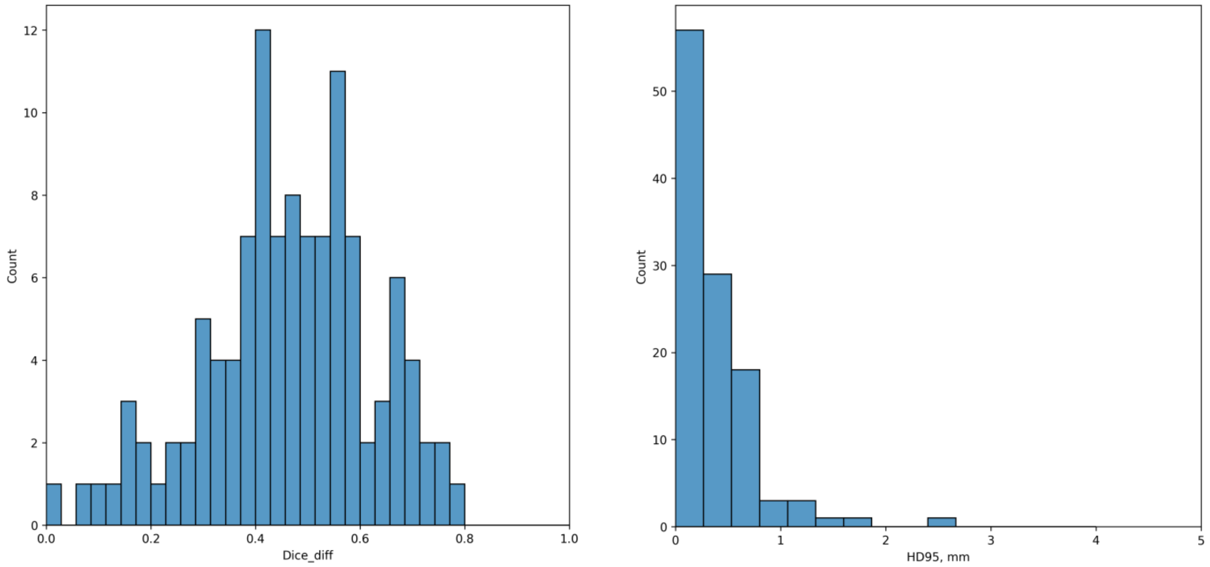

Supplementary Figure 5: Histogram of DSC and HD95 distribution of the deep learning model for GA growth area at month 12 in the external test set. DSC = Dice Similarity Coefficient, HD = Hausdorff distance, GA = geographic atrophy.

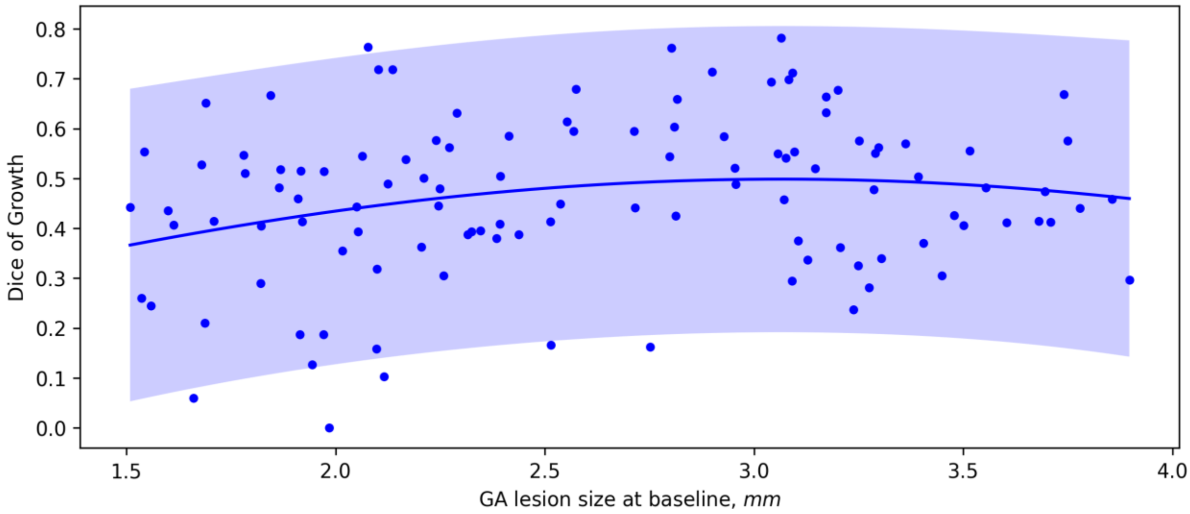

Supplementary Figure 6: Scatterplot for DSC distribution of the deep learning model for the GA growth areas across GA lesion sizes (square root transformed) at baseline. The blue stripe corresponds to the fitted gaussian process and indicates 95% limits of agreement. DSC = Dice Similarity Coefficient, GA = geographic atrophy.

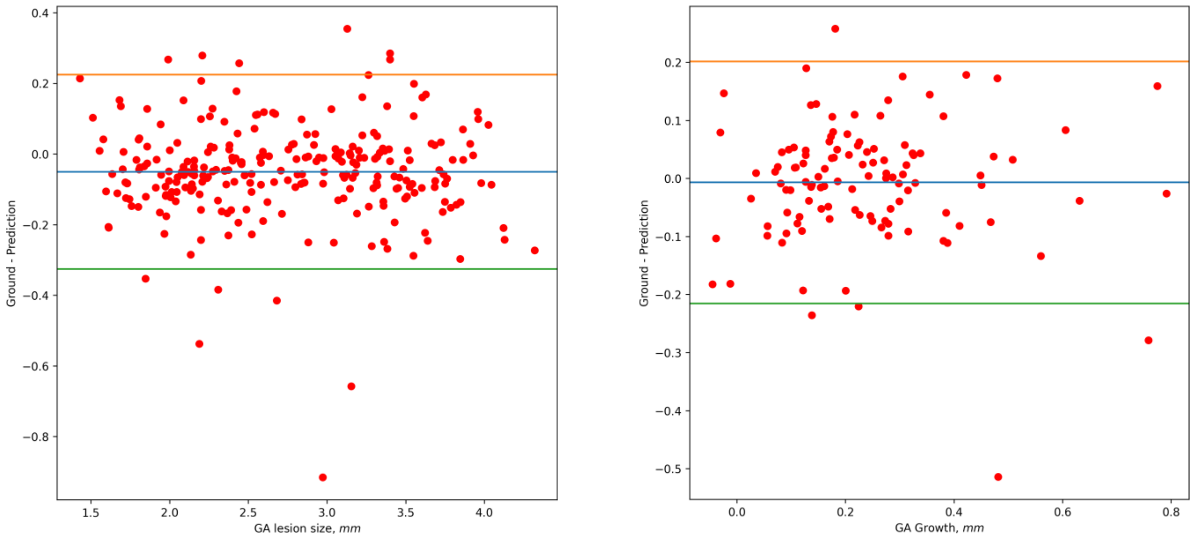

Supplementary Figure 7: Limits of agreement between manually annotated and predicted GA area by the deep learning model for baseline (left) and GA growth area at month 12 (right). GA = geographic atrophy.

Supplementary Tables

**Internal validation (MUV)**					
	N	Precision mean	Precision median, IQR	Recall mean	Recall median, IQR
Baseline	121	0.82 ± 0.19	0.89 [0.77;0.94]	0.86 ± 0.13	0.89 [0.83;0.94]
Month 12	121	0.87 ± 0.14	0.92 [0.84;0.96]	0.88 ± 0.1	0.90 [0.83;0.95]
All Scans	967	0.87 ± 0.15	0.92 [0.84;0.96]	0.87 ± 0.11	0.90 [0.84;0.94]
**External validation (FILLY)**					
	N	Precision mean	Precision median, IQR	Recall mean	Recall median, IQR
Baseline	113	0.90 ± 0.06	0.91 [0.87;0.94]	0.93 ± 0.06	0.94 [0.91;0.97]
Month 12	113	0.90 ± 0.06	0.92 [0.86;0.94]	0.94 ± 0.06	0.96 [0.92;0.97]
All Scans	226	0.90 ± 0.07	0.92 [0.87;0.94]	0.93 ± 0.06	0.95 [0.92;0.97]

Supplementary Table 1: Detailed evaluation metrics of the deep learning model for internal and external validation at baseline and month 12.
